# The impact of quantitative CT-based tumor volumetric features on the outcomes of patients with limited stage small cell lung cancer

**DOI:** 10.1186/s13014-020-1460-4

**Published:** 2020-01-14

**Authors:** Sophia C. Kamran, Thibaud Coroller, Nastaran Milani, Vishesh Agrawal, Elizabeth H. Baldini, Aileen B. Chen, Bruce E. Johnson, David Kozono, Idalid Franco, Nitish Chopra, Roman Zeleznik, Hugo J. W. L. Aerts, Raymond Mak

**Affiliations:** 1 Department of Radiation Oncology, Massachusetts General Hospital, Harvard Medical School, 55 Fruit Street, Cox 3, Boston, MA 02114 USA; 2 Harvard Medical School, Boston, MA USA; 3 Brigham and Women’s Hospital/Dana-Farber Cancer Institute, Harvard Medical School, 450 Brookline Avenue, Boston, MA 02215 USA; 4 MD Anderson Cancer Center, Houston, TX USA

**Keywords:** Small cell lung cancer, CT-based features, Tumor volume, Tumor diameter, Radiation, Chemoradiation

## Abstract

**Introduction:**

Limited stage small cell lung cancer (LS-SCLC) has a poor prognosis. Additional prognostic markers are needed for risk-stratification and treatment intensification. This study compares quantitative CT-based volumetric tumor measurements versus International Association for the Study of Lung Cancer (IASLC) TNM staging to predict outcomes.

**Materials & methods:**

A cohort of 105 patients diagnosed with LS-SCLC and treated with chemoradiation (CRT) from 2000 to 2013 were analyzed retrospectively. Patients were staged by the Union for International Cancer Control (UICC) TNM Classification, 8th edition. Tumor volumes and diameters were extracted from radiation planning CT imaging. Univariable and multivariable models were used to analyze relationships between CT features and overall survival (OS), locoregional recurrence (LRR), in-field LRR, any progression, and distant metastasis (DM).

**Results:**

Median follow-up was 21.3 months. Two-year outcomes were as follows: 38% LRR, 31% in-field LRR, 52% DM, 62% any progression, and 47% OS (median survival 16.5 months).

On univariable analysis, UICC T-stage and N-stage were not associated with any clinical outcome. UICC overall stage was only statistically associated with in-field LRR. One imaging feature (3D maximum tumor diameter) was found to be significantly associated with LRR (HR 1.10, *p* = 0.003), in-field LRR (HR 1.10, *p* = 0.007), DM (HR 1.10, *p* = 0.02), any progression (HR 1.10, *p* = 0.008), and OS (HR 1.10, *p* = 0.03). On multivariable analysis, this feature remained significantly associated with all outcomes.

**Conclusion:**

For LS-SCLC, quantitative CT-based volumetric tumor measurements were significantly associated with outcomes after CRT and may be better predictors of outcome than TNM stage.

## Introduction

There are projected to be 228,150 new cases of lung cancer and 142,670 deaths from lung cancer in 2019 in the United States [1]. Of these, approximately 10–15% of cases are characterized as small cell lung cancer (SCLC) [2]. Limited stage small cell lung cancer (LS-SCLC) makes up approximately 40% of all SCLC diagnoses [3], with a median overall survival of approximately 20 months [4–6].

Standard of care treatment for LS-SCLC is concurrent chest radiation plus chemotherapy [4, 7], but SCLC has a high propensity to recur within the radiation field and metastasize to distant sites. Given the persistent issues with both local control and distant recurrence, there is an urgent need to develop tools to identify patients at higher risk for early local recurrence or metastasis so more effective treatment approaches can be devised. Multiple clinical parameters have been previously identified to be associated with outcomes of patients with small cell lung cancer [8]. SCLC has historically been staged using the two-stage system, introduced by the Veterans’ Administration Lung Study Group [9], and is still in use in current clinical trials (NCT00632853). The International Association for the Study of Lung Cancer (IASLC) has previously suggested the incorporation of the Union for International Cancer Control (UICC) 7th edition tumor, node, metastasis (TNM) staging system into clinical practice for SCLC, given its strong prognostic significance in large national databases [10, 11]. However, there remains a paucity of data on its significance and validation in clinical practice, as well as validation of the updated 8th edition TNM staging system [12, 13]. Recently, quantitative tumor characteristics such as tumor diameter and tumor volume have been shown to have significant value in prognostication for non-small cell lung cancer (NSCLC), and tumor volume has been investigated as a biomarker in the management of localized and advanced NSCLC [14–20]. It is unclear whether these quantitative tumor characteristics, including tumor volume, obtained from imaging might similarly serve as prognostic biomarkers in SCLC.

In this study, we quantitatively analyzed CT-based volumetric assessments of LS-SCLC tumors at the time of radiation treatment planning to assess whether they correlated with outcome. We also characterized all LS-SCLC tumors per the UICC TNM Classification of Malignant Tumors 8th edition (2016) staging system. We compared TNM stage to quantitative “pre-radiation” tumor measurements to determine their utility as prognostic biomarkers with regard to clinical outcomes.

## Methods

### Patient selection

Under an IRB-approved protocol, patients with a diagnosis of LS-SCLC who were treated with radiotherapy +/− chemotherapy with curative intent at our institution were included in this study. A total of 105 patients were identified between 2000 and 2013 all of whom had CT imaging at the time of radiation simulation. Medical records were reviewed for patient, tumor, and treatment characteristics and clinical outcomes. Patients were excluded if they had documented metastases outside the chest radiotherapy field at the time of CT simulation.

### Tumor segmentation

Tumors were contoured on CT scans obtained at the time of CT simulation. Radiation planning CT scans (free-breathing) and tumor contours were retrieved from Eclipse Treatment Planning System (Varian, Palo Alto, CA). The gross tumor volume (GTV), including tumor and mediastinal nodes, was contoured on each slice for CT planning scans. All GTV contours were edited to exclude air, blood vessels, and normal tissue. All GTV contours were performed manually (S.C.K.) followed by subsequent approval by a separate radiation oncologist (R.H.M.).

### Volume calculations

Quantitative CT features including tumor volume, 2D axial maximal diameter, 2D coronal maximal diameter, 2D sagittal maximal diameter, and 3D maximal diameter were extracted from the GTV contours. 2D maximal diameter refers to the greatest diameter in either the axial/coronal/sagittal plane, while 3D maximal diameter refers to the greatest diameter in any direction. Quantitative measurement features were chosen based on ability to be practically measured in the clinic.

### Clinical endpoints

Patients were assessed for outcomes including locoregional recurrence (LRR), in-field LRR, distant metastases (DM), any progression, and overall survival (OS). Typical follow-up included chest CT scans every 3–4 months in the first 2 years after treatment completion and then every 6 months subsequently. LRR was defined as recurrence at or adjacent to the original tumor site, or in the hilar, mediastinal, or supraclavicular nodes. In-field LRR was defined as LRR within any part of the radiation field (in the planning target volume). All other sites were defined as DM. Any progression was defined as any LRR or DM. Time to LRR, in-field LRR, DM, and any progression were defined as the time interval from the end of treatment until the first radiographically-evident LRR, in-field LRR, or DM respectively. Patients were censored at the date of the last negative follow-up scans in patients without recurrence/metastases. OS was defined as the time from the end of treatment until death from any cause, censored at the last date of follow-up.

### Statistical analysis

All statistical analyses were performed using R version 3.3.2 [21]. Univariable Cox regression analyses and multivariable Cox regression analyses were built using backwards selection modeling (criteria *p* = 0.2). Clinically relevant variables were used to identify clinical or imaging features associated with the outcomes of LRR, in-field LRR, DM, any progression, or OS. The Kaplan-Meier method was used to generate actuarial survival estimates and plots for local control, progression-free survival, and OS. Pearson correlation was used to compare potential predictors. *P*-values were considered significant for less than 0.05.

## Results

### Patient and tumor characteristics

There were 105 patients with LS-SCLC who received radiation therapy included in the analysis. All patients underwent simulation CT scan for radiation planning. Patient and tumor characteristics are listed in Table 1**.** The cohort consisted of more men than women (60% male) with a median age at diagnosis of 64 years (range, 44–88). The majority of patients had a performance score (PS) of 0 or 1 (81%) and were current/former smokers (97%) with median pack-years of 45 (range, 1.3–127.5). Per UICC staging, 31% (*n* = 33) were stage IIIA, 39% (*n* = 41) were stage IIIB, and 21% (*n* = 22) were stage IIIC. The median tumor volume in all 105 patients at the time of CT simulation was 48.5 cm^3^ (range 0.2–428), the median axial tumor diameter was 7.4 cm (range 1.0–15.1), and the median maximum 3D tumor diameter was 10.8 cm (range 1.0–22.2). When comparing different measurement predictors, tumor volume and maximum 3D tumor diameter only mildly correlated with each other (*R* = 0.49, Pearson correlation).

**Table 1:** Patient and tumor characteristics for patients with limited stage small cell lung cancer (LS-SCLC) treated with chemoradiation, (*n* = 105)

Characteristic	*N* = 105 (%)
Gender	
Female	42 (40%)
Male	63 (60%)
Age	
Median (range)	63.8 (range 44.4–87.5)
≥60	71 (68%)
< 60	34 (32%)
Race	
White	93 (89%)
African American	6 (6%)
Asian	1 (1%)
Not available/other	5 (5%)
Performance score	
0	32 (30%)
1	53 (51%)
2	15 (14%)
3	4 (4%)
Unknown	1 (1%)
Smoking History	
Current smoker at time of dx of < 1 year prior	56 (53%)
Former smoker (quit > 1 year prior)	46 (44%)
Never smoker	3 (3%)
Pack-years	
Median (range)	45 (1.3–127.5)
T stage	
T0	3 (3%)
T1	28 (27%)
T2	12 (11%)
T3	27 (26%)
T4	35 (33%)
N stage	
N0	6 (6%)
N1	11 (10%)
N2	55 (52%)
N3	33 (31%)
Overall stage	
IA1	1 (1%)
IA3	2 (2%)
IIB	6 (6%)
IIIA	33 (31%)
IIIB	41 (39%)
IIIC	22 (21%)
Tumor volume (cm^3^)	
Median (range)	48.5 (0.2–428)
Maximum 2D tumor diameter axial (cm)	
Median (range)	7.4 (1.0–15.1)
Maximum 2D tumor diameter coronal (cm)	
Median (range)	8.6 (0.6–21.6)
Maximum 2D tumor diameter sagittal (cm)	
Median (range)	8.30 (0.9–20.7)
Maximum 3D tumor diameter (cm)	
Median (range)	10.8 (1.0–22.2)

### Treatment characteristics

Of the 105 patients, 92 (88%) received concurrent chemotherapy with chest radiation, while 13 patients (12%) received radiation alone without any chemotherapy (Table 2). The majority of the chemotherapy was cisplatin/etoposide. The median number of chemotherapy cycles was 3 (range, 2–7). Eighty-one percent of patients received induction chemotherapy, which was defined as at least one cycle of chemotherapy received before the initiation of radiation. Median RT dose received to the thorax was 45 Gy (range, 20–66.6), with approximately half of the population receiving twice daily (BID) treatment. All patients who received BID treatment received 45 Gy, while those who received daily fractionation received a median dose of 60 Gy (range, 20–66.6). Prophylactic cranial irradiation (PCI) was given to 60% of patients.

**Table 2:** Treatment characteristics for patients with limited stage small cell lung cancer (LS-SCLC) treated with chemoradiation, (*n* = 105)

Treatment	*N* = 105 (%)
Induction chemotherapy	85 (81%)
Concurrent chemoradiation	92 (88%)
Radiation alone	13 (12%)
Post-radiation chemotherapy	53 (50%)
Any chemotherapy	102 (97%)
Chemotherapy type	
Cisplatin/etoposide	79
Carboplatin/etoposide	11
Carboplatin/taxol	1
Cisplatin/irinotecan	1
Not available	2
Median number of chemotherapy cycles (range)	3 (2–7)
PCI^a^	
Yes	63 (60%)
No	42 (40%)
Median RT dose received (range)	45 (20–66.6)
Daily	52 (49%)
BID	51 (49%)
Not available	2 (2%)

^a^*PCI* prophylactic cranial irradiation

### Locoregional recurrence, distant metastases, any progression, and overall survival

**Fig. 1:**
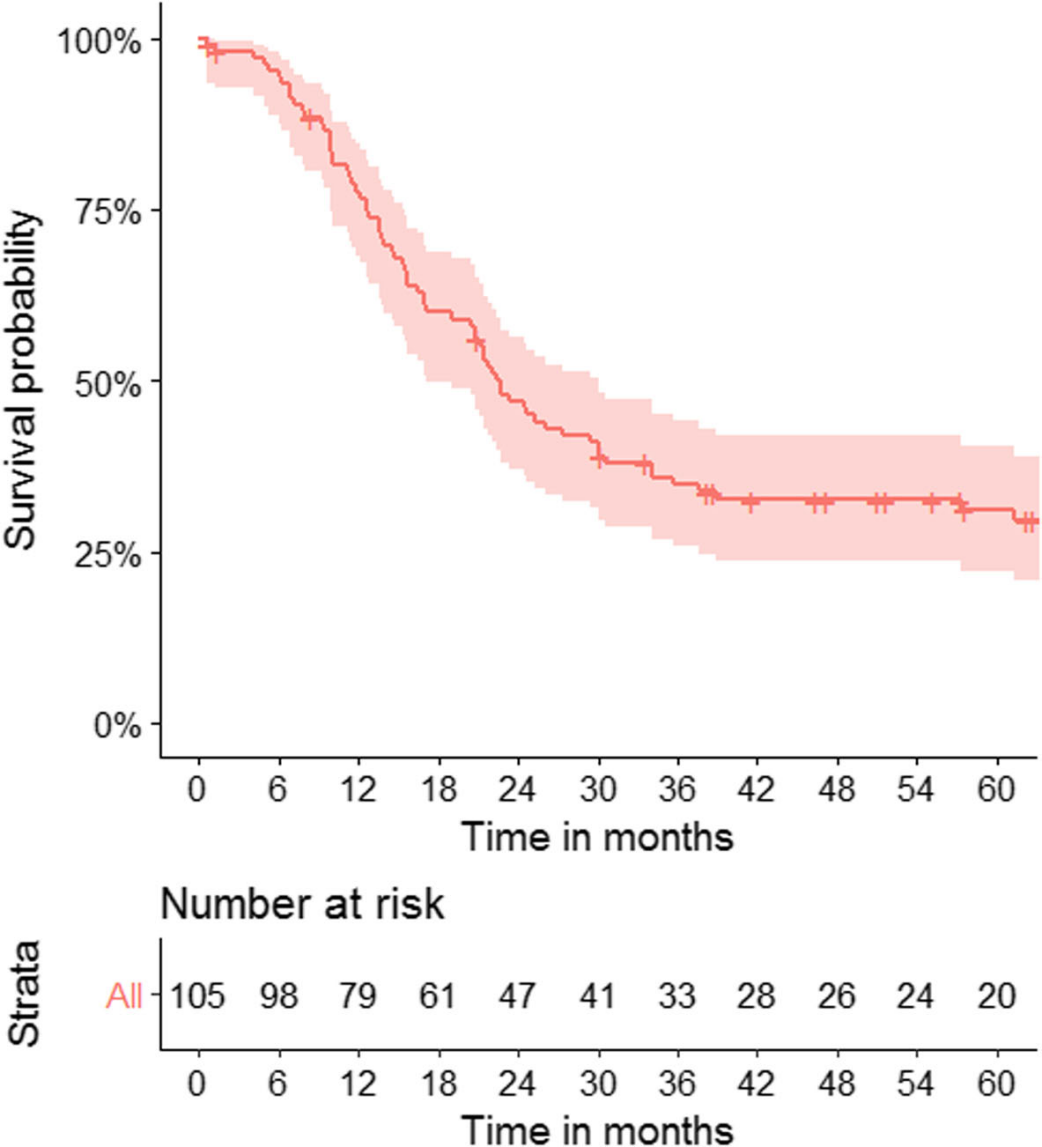
Kaplan-Meier plot of overall survival. Median follow-up was 21.3 months (range, 0.6–113.4), and two-year OS was 47%

Median follow-up was 21.3 months (range, 0.6–113.4). Two-year LRR rate was 38%, and median time to LRR was 8.6 months (range, 2.5–47.3) (Table 3). Two-year in-field LRR was 31%, and median time to in-field LRR was 8.5 months (range, 2.5–47.3). Two-year DM rate was 52% with median time to DM of 8.8 months (range, 1.6–74.5). Two-year any progression rate was 62%, with median time to progression of 8.4 months (range, 1.6–26.3). Median survival was 21.7 months, with a 2-year OS of 47% (Fig. 1).

**Table 3:** Locoregional recurrence, distant metastasis, and survival outcomes for patients with limited stage small cell lung cancer (LS-SCLC) treated with chemoradiation, (*n* = 105)

Outcomes	Rate
Two-year LRR^a^	38%
Two-year in-field LRR rate	31%
Median time to LRR (months, range)	8.6 (2.5–47.3)
Median time to in-field LRR (months, range)	8.5 (2.5–47.3)
Two-year DM^b^ rate	52%
Median time to DM (months)	8.8 (1.6–74.5)
Two-year any progression rate	62%
Median time to any progression (months, range)	8.4 (1.6–26.2)
Two-year OS^c^ rate	47%
Median survival (months, range)	16.5 (0.6–98.6)

^a^*LRR* loco-regional recurrence, ^b^*DM* distant metastasis, ^c^*OS* overall survival

### Predictors of outcomes: univariable and multivariable analyses

#### Locoregional recurrence

On univariable analysis, the axial maximum 2D tumor diameter, coronal maximum 2D tumor diameter, sagittal maximum 2D tumor diameter, and maximum 3D tumor diameter were significantly associated with increased risk for LRR (all HRs 1.10, *p* = 0.003–0.02) (Table 4). In the multivariable adjusted model, the use of concurrent chemotherapy was significant for decreased risk of LRR (HR 0.21, 95%CI 0.07–0.66, *p* = 0.01), and increased maximum 3D tumor diameter remained significant for increased risk of LRR (HR 1.20, 95%CI 1.10–1.30, *p* = 0.001) as well as increased RT fractionation (HR 2.08, 95%CI 1.03–4.23, *p* = 0.04) (Table 5).

**Table 4:** Univariate Analysis of predictors for Loco-regional recurrence (LRR), distant metastasis (DM), any progression and overall survival (OS) for patients with limited stage small cell lung cancer (LS-SCLC) treated with chemoradiation, (*n* = 105)

Category	LRR		In-field LRR		DM		Any progression		OS	
	HR (95% CI)	*p*-value	HR (95% CI)	*p*-value	HR (95% CI)	*p*-value	HR (95% CI)	*p*-value	HR (95% CI)	*p*-value
Gender										
Female^a^	–	–	–	–	–	–	–	–	–	–
Male	0.75 (0.39–1.44)	0.39	0.80 (0.38–1.71)	0.57	0.70 (0.41–1.21)	0.20	0.70 (0.43–1.16)	0.17	0.50 (0.32–0.80)	**0.003**^c^
Age (continuous)	0.97 (0.94–1.01)	0.20	0.95 (0.91–0.99)	**0.04**	1.00 (0.97–1.03)	0.96	0.99 (0.96–1.02)	0.56	1.01 (0.98–1.04)	0.46
Race										
White^a^	–	–	–	–	–	–	–	–	–	–
African American	0.65 (0.09–4.76)	0.67	–	0.99	1.44 (0.45–4.64)	0.54	1.22 (0.38–3.89)	0.74	1.31 (0.47–3.66)	0.61
	0.37 (0.05–2.67)				0.89 (0.28–2.86)		0.71 (0.22–2.28)		1.00 (0.36–2.76)	
Other^b^		0.32	0.47 (0.06–3.74)	0.46		0.84		0.60		0.99
Performance score										
0^a^	–	–	–	–	–	–	–	–	–	–
≥1	2.00 (0.94–4.25)	0.07	1.61 (0.71–3.66)	0.25	2.92 (1.46–5.84)	**0.002**^c^	2.90 (1.53–5.48)	**0.001**^c^	3.05 (1.70–5.45)	**< 0.001**^c^
Smoking history										
Current^a^	–	–	–	–	–	–		–	–	–
Former	0.71 (0.36–1.39)	0.32	–	–	1.05 (0.61–1.79)	0.87	0.99 (0.60–1.65)	0.97	1.15 (0.72–1.82)	0.56
	1.92 (0.45–8.19)				0.52 (0.07–3.83)		1.16 (0.28–4.81)		0.99 (0.24–4.1)	
Never		0.38	–	–		0.52		0.84		0.99
Pack-years	1.01 (0.99–1.02)	0.21	1.00 (0.99–1.02)	0.96	1.01 (1.00–1.02)	0.11	1.01 (0.99–1.01)	0.20	1.01 (1.00–1.02)	0.12
T stage										
T0–1^a^	–	–	–	–	–	–	–	–	–	–
T2–4	0.66 (0.34–1.28)	0.22	0.93 (0.44–1.99)	0.85	0.64 (0.37–1.12)	0.12	0.64 (0.38–1.08)	0.09	0.67 (0.41–1.10)	0.11
N stage										
N0–1^a^	–	–	–	–	–	–	–	–	–	–
N2–3	5.47 (0.75–39.93)	0.09	–	–	1.64 (0.59–4.56)	0.34	2.06 (0.75–5.67)	0.16	1.35 (0.62–2.95)	0.45
Overall stage										
IIIB^a^	–	–	–	–	–	–	–	–	–	–
IA-IIIA	0.59 (0.28–1.25)	0.17	0.32 (0.13–0.78)	**0.01**^**c**^	0.68 (0.36–1.28)	0.23	0.69 (0.39–1.22)	0.20	0.73 (0.44–1.22)	0.23
	1.34		0.33 (0.12–0.94)		1.45 (0.75–2.78)		1.37 (0.73–2.55)		0.97 (0.54–1.79)	
	(0.61–2.95)									
IIIC		0.47		**0.04**		0.26		0.33		0.95
Induction chemotherapy										
Yes	1.77 (0.69–4.53)	0.24	1.67 (0.58–4.80)	0.34	1.33 (0.65–2.73)	0.43	1.43 (0.73–2.82)	0.30	1.65 (0.87–3.14)	0.12
No^a^	–	–	–		–	–	–	–	–	–
Concurrent chemotherapy										
Yes	0.43 (0.17–1.11)	0.08	0.89 (0.21–3.77)	0.88	0.40 (0.18–0.90)	**0.03**	0.33 (0.16–0.68)	**0.003**^c^	0.29 (0.15–0.54)	**< 0.001**^c^
No^a^	–		–	–	–		–	–	–	–
PCI										
Yes	0.71 (0.37–1.36)	0.30	1.21 (0.53–2.74)	0.65	0.40 (0.23–0.68)	**< 0.001**^c^	0.44 (0.27–0.73)	**0.001**^c^	0.37 (0.23–0.59)	**< 0.001**^c^
No^a^	–	–	–	–	–	–	–	–	–	–
Median RT dose	0.97 (0.93–1.01)	0.15	0.94 (0.90–0.99)	**0.02**^c^	1.03 (1.00–1.06)	**0.04**	1.01 (0.99–1.04)	0.34	1.01 (0.99–1.04)	0.34
RT fractionation										
Daily^a^	–	–	–	–	–	–	–	–	–	–
BID	1.13 (0.59–2.13)	0.72	1.49 (0.70–3.16)	0.30	0.68 (0.40–1.17)	0.16	0.76 (0.46–1.26)	0.29	0.64 (0.40–1.02)	0.06
Tumor volume (cm^3^)	1.00 (1.00–1.00)	0.21	1.00 (1.00–1.00)	0.11	1.00 (1.00–1.00)	0.61	1.00 (1.00–1.00)	0.23	1.00 (1.00–1.00)	0.90
Maximum 2D tumor diameter axial (cm)	1.10 (1.00–1.20)	**0.02**^c^	1.10 (0.90–1.20)	0.08	1.10 (0.90–1.20)	0.09	1.10 (0.90–1.20)	0.05	1.10 (0.90–1.10)	0.14
Maximum 2D tumor diameter coronal (cm)	1.10 (1.00–1.20)	**0.01**^c^	1.10 (1.00–1.20)	**0.008**^c^	1.10 (1.00–1.20)	**0.03**	1.10 (1.00–1.10)	**0.02**^c^	1.10 (0.90–1.10)	0.11
Maximum 2D tumor diameter sagittal (cm)	1.10 (1.00–1.20)	**0.01**^c^	1.10 (1.00–1.20)	**0.01**^c^	1.10 (0.90–1.10)	0.14	1.10 (0.90–1.10)	0.10	1.00 (0.90–1.10)	0.38
Maximum 3D tumor diameter (cm)	1.10 (1.00–1.20)	**0.003**^c^	1.10 (1.00–1.20)	**0.007**^c^	1.10 (1.00–1.20)	**0.02**	1.10 (1.00–1.10)	**0.008**^c^	1.10 (1.00–1.20)	**0.03**

^a^reference value; ^b^ Asian, not available and other; Bolded values indicate significance, *p*<0.05

^c^Meets significance after adjustment for hypothesis testing using a Benjamini-Hochberg FDR of 0.1

**Table 5:** Multivariable Cox Analysis of predictors for Loco-regional recurrence (LRR), distant metastasis (DM), any progression and overall survival (OS) for patients with limited stage small cell lung cancer (LS-SCLC) treated with chemoradiation, (*n* = 105)

	LRR	
	HR (95% CI)	*p*-value
RT fractionation	2.08 (1.03–4.23)	***0.04***^*c*^
Maximum 3D tumor diameter (cm)	1.20 (1.10–1.30)	***< 0.001***^*c*^
Pack-years	1.01 (1.00–1.02)	0.10
Concurrent chemotherapy	0.24 (0.08–0.69)	***0.008***^*c*^
	**In-field LRR**	
	**HR (95% CI)**	***p*-value**
Maximum 3D tumor diameter (cm)	1.20 (1.01–1.40)	***0.02***^*c*^
Tumor Volume	1.01 (1.00–1.01)	***0.03***^*c*^
Maximum 2D tumor diameter sagittal (cm)	0.95 (0.94–1.01)	0.08
Maximum 2D tumor diameter axial (cm)	0.95 (0.95–1.01)	0.08
	**DM**	
	**HR (95% CI)**	***p*-value**
T stage^b^	0.58 (0.32–1.05)	0.07
Performance status ≥1	2.54 (1.27–5.09)	***0.009***^*c*^
PCI		
Yes	0.61 (0.34–1.09)	0.10
Pack-years	1.01 (1.00–1.02)	0.12
Median RT dose	1.03 (1.00–1.06)	0.09
Maximum 3D tumor diameter (cm)	1.10 (1.01–1.10)	***0.03***^*c*^
	**Any progression**	
	**HR (95% CI)**	***p*-value**
Performance status ≥1	2.47 (1.31–4.67)	***0.005***^*c*^
PCI		
Yes	0.57 (0.34–0.95)	***0.03***^*c*^
T stage^b^	0.61 (0.35–1.05)	0.07
Maximum 3D tumor diameter (cm)	1.10 (1.01–1.10)	***0.01***^*c*^
	**OS**	
	**HR (95% CI)**	***p*-value**
Overall stage^a^	1.09 (0.58–2.07)	0.78
T stage_b_	0.53 (0.31–0.90)	***0.02***^*c*^
Gender		
Male	0.34 (0.20–0.56)	***< 0.001***^*c*^
Performance status ≥1	3.16 (1.71–5.85)	***< 0.001***^*c*^
RT fractionation	0.58 (0.35–0.97)	***0.04***^*c*^
Concurrent chemotherapy	0.43 (0.21–0.89)	***0.02***^*c*^
Maximum 3D tumor diameter (cm)	1.10 (0.99–1.10)	0.09

^**a**^Stage IA-IIIB as referent

^b^T stage 0–1 as referent; Bolded, italicized values indicate significance, *p*<0.05

^c^Meets significance after adjustment for hypothesis testing using a Benjamini-Hochberg FDR of 0.1

#### In-field locoregional recurrence

On univariable analysis of in-field LRR, age (HR 0.95, *p* = 0.04), overall stage of IA-IIIA (HR 0.32, *p* = 0.01) or IIIC (HR 0.33, *p* = 0.04), and median RT dose (HR 0.94, *p* = 0.02) were significantly associated with a decreased risk for in-field LRR, while coronal maximum 2D diameter, sagittal maximum 2D diameter, and maximum 3D tumor diameter were all significantly associated with an increased risk for in-field LR (all HRs 1.10, *p* = 0.007–0.01). On multivariable, adjusted analysis, larger maximum 3D tumor diameter remained significantly associated with an increased risk for in-field LRR (HR 1.20, 95%CI 1.01–1.40; *p* = 0.02), as well as tumor volume (HR 1.01, 95%CI 1.00–1.01, *p* = 0.03).

### Distant metastases

On univariable analysis, increased maximum 3D tumor diameter (HR 1.10, *p* = 0.02) and coronal maximum 2D tumor diameter (HR 1.10, *p* = 0.03), PS ≥1 (HR 2.92, *p* = 0.002), median RT dose (HR 1.03, *p* = 0.04), and PS ≥1 (HR 2.92, *p* = 0.002) were variables significantly associated with a higher risk for DM, while the use of PCI (HR 0.40, *p* < 0.001) and concurrent chemotherapy (HR 0.40, *p* = 0.03) were significantly associated with a lower risk for DM. On multivariable analysis, PS ≥1 (HR 2.54, 95% CI 1.27–5.09, *p* = 0.009) and maximum 3D tumor diameter (HR 1.10, 95% CI 1.01–1.10, *p* = 0.03) were significant for higher risk of DM.

### Any progression

PS ≥1 (HR 2.90, *p* = 0.001), coronal maximum 2D diameter (HR 1.10, *p* = 0.02), and maximum 3D tumor diameter (HR 1.10, *p* = 0.008) were all significantly associated with any progression on univariable analysis, while treatment with PCI (HR 0.44, *p* = 0.001) and concurrent chemotherapy (HR 0.33, *p* = 0.003) were significantly associated with a decreased risk of any progression. In multivariable analysis, PS ≥1 (HR 2.47, 95% CI 1.31–4.67, *p* = 0.005) and maximum 3D tumor diameter (HR 1.10, 95% CI 1.01–1.10, *p* = 0.01) were significantly associated with any progression, while PCI was significantly associated with a decreased risk of any progression (HR 0.57, 95% CI 0.34–0.95, *p* = 0.03).

### Overall survival

For overall survival, larger maximum 3D tumor diameter (HR 1.10, *p* = 0.03) and PS ≥1 (HR 3.05, *p* < 0.001) were significantly associated with a higher risk of death, while use of PCI (HR 0.37, *p* < 0.001), concurrent chemotherapy (HR 0.29, *p* < 0.001), and male gender (HR 0.50, *p* = 0.003) were associated with a lower risk of death. In the multivariable model, also forcing in overall stage, PS ≥1 remained as a significant determinant of worse OS (HR 3.16, 95%CI 1.71–5.85, *p* < 0.001) while male gender (HR 0.34, 95% CI 0.20–0.56, *p* < 0.001), increased RT fractionation (HR 0.58, 95% CI 0.35–0.97, *p* = 0.04), concurrent chemotherapy with chest RT (HR 0.43, 95% CI 0.21–0.80, *p* = 0.02) and T stage 2–4 (HR 0.53, 95% CI 0.31–0.90, *p* = 0.02) led to a significantly decreased risk of death. In the adjusted model, increasing maximum 3D tumor diameter trended toward a worsened OS (*p* = 0.09). To attempt to account for any potential confounding of receipt of PCI on OS, additional analyses were performed (Additional file 1: Tables S1-3) which did not yield any significant differences. Attempts to analyze continuous volumetric features as categorial variables were also not successful due to small numbers of patients/events (Additional file 1: Table S4).  

## Discussion

In this study, we evaluated the utility of using quantitative “pre-radiation” CT-based variables related to tumor volume and diameter as prognostic biomarkers for outcomes, in addition to other clinical variables, including the UICC TNM 8th edition staging system, in limited stage small cell lung cancer. We found that CT-based volumetric variables, including maximum 3D tumor diameter, were associated with outcomes including locoregional recurrence, in-field LRR, distant metastases, any progression, and overall survival. We did not find any significant associations between T stage, N stage, or overall UICC stage and LRR, DM, any progression, or OS; overall UICC stage was only significantly associated with in-field LRR on univariate analysis. On multivariable analysis, maximum 3D tumor diameter continued to perform as a significant prognostic factor for LRR, in-field LRR, DM and any progression.

The goal of clinical staging is for it to be defined such that it is associated with prognosis and can therefore help guide treatment strategy. SCLC has historically been staged as “limited” or “extensive,” which was introduced by the Veterans’ Administration Lung Study Group [9]. Recently, the applicability of TNM staging to SCLC has been advocated. Yet, the TNM prognostic ability has been questioned, particularly in non-surgical series, as it had been shown to be prognostic of outcome in small, single-institution surgical series [22–24]. Shepherd, et al. tested the prognostic ability of the 7th edition TNM system in the International Association for the Study of Lung Cancer (IASLC) database, finding 8088 SCLC patients with enough data to classify into TNM stage [10]. In looking at survival only, it was found that survival directly correlated with the T and N stage. However, this study did not examine any other outcomes such as recurrence, nor did it have information on clinical treatment. In a different database study, Ou, et al. compared the 7th edition TNM system to the previous 6th edition using 10,660 SCLC patients from the California Cancer Registry, finding that stage groupings performed better in separation of survival curves among those with early stage SCLC compared to the prior edition [11]. Yet, this database study similarly lacked treatment data, as well as information on recurrences or distant metastases. In a validation of the 8th edition TNM system, Abdel-Rahman used the SEER database to evaluate both the 7th and 8th edition for SCLC patients, finding that both the 7th and 8th edition performed better than the prior Veterans’ Administration system with respect to prognostic ability for cancer-specific and overall survival, but with modest improvement for the 8th compared to the 7th in patients with SCLC [13]. Jhun, et al. examined the TNM stage in a clinical cohort of 320 SCLC patients [12]. Approximately 28% of their patient population underwent definitive concurrent chemoradiation, with the majority of their patients (~ 70%) receiving palliative treatment. The median OS in this cohort was 12.5 months. It was found that T stage was not a significant predictor for OS, but that N and M stage variables were significant. In our series of LS-SCLC patients treated predominantly with chemoradiation, we found that overall stage corresponded with in-field LRR, but this association did not hold up on the multivariable adjusted analysis. Overall and N stage did not correspond with any other outcome. T stage was significantly associated with OS in multivariable analysis, but not with any other outcome. We did not include metastatic patients at diagnosis, and our study included other outcomes of interest for SCLC, namely recurrence and distant metastases. The majority of patients in this chemoradiation series were AJCC 8th edition stage IIIA, IIIB, or IIIC as expected, and the lack of association between outcomes and IIIA versus IIIB versus IIIC does bring into question the suitability of applying this NSCLC staging system to LS-SCLC. However, our finding of a lack of TNM association with outcomes could be related to the small sample size, therefore underpowering our associations for statistical significance. In our data, we do observe a trend of higher N stage (N stage 2–3) associating with poorer outcomes, but this did not reach statistical significance.

CT-based features, including tumor volume, have been found to correlate with outcomes for NSCLC. Su, et al. found that tumor volume contributed significantly as an independent prognostic factor for disease-free survival and OS in stage I NSCLC [14]. In a separate study in stage III NSCLC patients, it was found that the GTV at the time of radiation planning was independently associated with survival [17, 20, 25]. In our study, we found that the maximum 3D tumor diameter (in any plane) at the time of radiation planning correlates best with outcomes, compared to tumor volume. This could be related to the tendency for SCLC to present with largely irregular tumors that can be bulky in multiple planes, which might contribute more to outcome than the volume of tumor itself, especially as SCLC is highly responsive to chemoradiation and volume can be dramatically reduced over a short period of time. Outcome may be related more to how the tumor spreads, rather than the overall volume. Tumor volume and maximum 3D tumor diameter only mildly correlated with each other. Nevertheless, to our knowledge, such CT-based quantitative tools relating to SCLC have not yet been investigated, and this is the first such rigorous investigation. These findings open the possibility for adding additional clinical tools to the current known prognostic factors that can further aid our early stratification of LS-SCLC patients.

This study must be interpreted in the context of several limitations. The study is retrospective in nature, and therefore subject to inherent biases. Tumor volume was obtained at the time of CT simulation, after many of the patients received 1–2 cycles of chemotherapy, and there may be greater predictive power with earlier time points. However, on subgroup analysis, maximum 3D tumor diameter was still significant among those who received induction chemotherapy and among those who did not (data not shown). In addition, we are not looking at volume as a predictive biomarker in this study, but rather treating the “pre-radiation” tumor volume as a prognostic biomarker. Several of the measurement features (e.g. 3D maximum tumor diameter) can be subject to observer variability given the complex shapes of tumor volumes. Finally, the sample size is small, resulting in insufficient power, thus the findings from this study will require validation in a larger and/or external cohort.

Despite these limitations, this is the first study to demonstrate that CT-based quantitative features have significant correlation with outcomes in patients with LS-SCLC. Given that SCLC patients continue to have a poor prognosis, it is imperative to find early biomarkers that can reliably predict outcome, as patients at high-risk for recurrence or distant metastases could be considered for treatment intensification.

## Conclusion

In summary, this study demonstrates a significant association between quantitative CT-based tumor features and outcomes in limited stage small cell lung cancer. Our study demonstrates that these features may be a useful predictor of outcome for limited stage SCLC. The quantitative CT-based variables need to be further validated in larger clinical cohorts but demonstrate promise in the era of precision medicine for SCLC.

## Supplementary information

**Additional file 1: Table S1.** Univariate Analysis of predictors for Loco-regional recurrence (LRR), distant metastasis (DM), any progression and overall survival (OS) for patients with limited stage small cell lung cancer (LS-SCLC) treated with prophylactic cranial irradiation, (*n* = 63). **Table S2.** Multivariable Cox Analysis of predictors for Loco-regional recurrence (LRR), distant metastasis (DM), any progression and overall survival (OS) for patients with limited stage small cell lung cancer (LS-SCLC) treated with prophylactic cranial irradiation, (*n* = 63). **Table S3.** Univariate Analysis of predictors for Loco-regional recurrence (LRR), distant metastasis (DM), any progression and overall survival (OS) for patients with limited stage small cell lung cancer (LS-SCLC) treated without prophylactic cranial irradiation, (*n* = 42). **Table S4.** Univariate Analysis of predictors for Loco-regional recurrence (LRR), distant metastasis (DM), any progression and overall survival (OS) for patients with limited stage small cell lung cancer (LS-SCLC) treated with chemoradiation, (*n* = 105).

## Data Availability

The datasets during and/or analysed during the current study available from the corresponding author on reasonable request.

## Abbreviations

CRT: Chemoradiation
DM: Distant metastasis
GTV: Gross tumor volume
IASLC: International Association for the Study of Lung Cancer
LRR: Locoregional recurrence
LS-SCLC: Limited stage small cell lung cancer
NSCLC: Non-small cell lung cancer
OS: Overall survival
PCI: Prophylactic cranial irradiation
SCLC: Small cell lung cancer
TNM: Tumor, node, metastasis
UICC: Union for International Cancer Control
