# Supplementary material for: The impact of quantitative CT-based tumor volumetric features on the outcomes of patients with limited stage small cell lung cancer
Source: Radiat Oncol. 2020 Jan 14;15:14. doi: 10.1186/s13014-020-1460-4 (PMC6961251; doi:10.1186/s13014-020-1460-4)
Supplement: Supplementary file 1 — Additional file 1: Table S1. Univariate Analysis of predictors for Loco-regional recurrence (LRR), distant metastasis (DM), any progression and overall survival (OS) for patients with limited stage small cell lung cancer (LS-SCLC) treated with prophylactic cranial irradiation, (n = 63). Table S2. Multivariable Cox Analysis of predictors for Loco-regional recurrence (LRR), distant metastasis (DM), any progression and overall survival (OS) for patients with limited stage small cell lung cancer (LS-SCLC) treated with prophylactic cranial irradiation, (n = 63). Table S3. Univariate Analysis of predictors for Loco-regional recurrence (LRR), distant metastasis (DM), any progression and overall survival (OS) for patients with limited stage small cell lung cancer (LS-SCLC) treated without prophylactic cranial irradiation, (n = 42). Table S4. Univariate Analysis of predictors for Loco-regional recurrence (LRR), distant metastasis (DM), any progression and overall survival (OS) for patients with limited stage small cell lung cancer (LS-SCLC) treated with chemoradiation, (n = 105). [file 13014_2020_1460_MOESM1_ESM.docx]

**Supplemental Table 1.** **Univariate Analysis of predictors for Loco-regional recurrence (LRR), distant metastasis (DM), any progression and overall survival (OS) for patients with limited stage small cell lung cancer (LS-SCLC) treated with prophylactic cranial irradiation, (n=63)**

| **Category** | **LRR** | | **In-field LRR** | | **DM** | | **Any progression** | | **OS** | |
| --- | --- | --- | --- | --- | --- | --- | --- | --- | --- | --- |
|  | **HR (95% CI)** | **p-value** | **HR (95% CI)** | **p-value** | **HR (95% CI)** | **p-value** | **HR (95% CI)** | **p-value** | **HR (95% CI)** | **p-value** |
| Gender  Female*  Male | -  0.41 (0.18-0.93) | -  **0.03** | -  0.53 (0.22-1.30) | -  0.17 | -  0.52 (0.24-1.10) | -  0.09 | -  0.45 (0.23-0.88) | -  **0.02** | -  0.38  (0.20-0.73) | -  **0.004^#^** |
| Age (continuous) | 0.94 (0.89-0.99) | **0.04** | 0.97 (0.92-1.03) | 0.37 | 0.99 (0.94-1.05) | 0.74 | 0.96 (0.92-1.01) | 0.16 | 0.99 (0.94-1.03) | 0.47 |
| Race  White*  African American  Other^$^ | -  -  - | -  0.99  0.99 | -  -  - | -  0.99  0.99 | -  1.13 (0.15-8.41)  1.22 (0.17-9.00) | -  0.91  0.85 | -  0.96 (0.13-7.02)  0.87 (0.12-6.37) | -  0.97  0.89 | -  1.28 (0.29-5.57)  1.05 (0.14-7.68) | -  0.75  0.96 |
| Performance score  0*  ≥1 | -  2.08 (0.85-5.08) | -  0.11 | -  3.88 (1.09-13.87) | -  **0.04** | -  2.38 (1.05-5.40) | -  **0.04** | -  2.31 (1.10-4.84) | -  **0.03** | -  2.51 (1.25-5.04) | -  **0.009^#^** |
| Smoking history  Current*  Former  Never | -  1.05 (0.43-2.52)  2.59 (0.58-11.56) | -  0.92  0.21 | -  1.90 (0.69-4.99)  3.40 (0.67-17.38) | -  0.22  0.14 | -  1.16 (0.55-2.44)  0.75 (1.00-5.71) | -  0.71  0.79 | 1.14 (0.56-2.31)  1.72 (0.40-7.41) | -  0.71  0.47 | -  1.20 (0.62-2.32)  1.45 (0.34-6.23) | -  0.58  0.61 |
| Pack-years | 1.01 (0.99-1.02) | 0.47 | 0.98 (0.97-1.01) | 0.14 | 1.01 (0.99-1.03) | 0.06 | 1.01 (0.99-1.02) | 0.17 | 1.01 (1.00-1.03) | **0.04** |
| T stage  T0-1*  T2-4 | -  0.66 (0.26-1.57) | -  0.33 | -  2.60 (0.94-7.23) | -  0.07 | -  0.65 (0.29-1.47) | -  0.31 | -  0.72 (0.34-1.55) | -  0.40 | -  0.70 (0.33-1.44) | -  0.33 |
| N stage  N0-1*  N2-3 | -  - | -  0.99 | -  - | -  - | -  1.72 (0.41-7.25) | -  0.46 | -  2.28 (0.55-9.52) | -  0.26 | -  1.63 (0.50-5.32) | -  0.42 |
| Overall stage  IIIB*  IA-IIIA  IIIC | -  -  - | -  -  - | -  -  2.18 (0.83-5.76) | -  **^-^**  0.11 | -  1.59 (0.35-7.12)  1.68 (0.78-3.85) | -  0.55  0.18 | -  1.12 (0.26-4.87)  1.40 (0.70-2.81) | -  0.88  0.34 | -  0.72 (0.16-3.13)  1.07 (0.56-2.06) | -  0.66  0.84 |
| Induction chemotherapy  Yes  No* | 2.23 (0.66-7.52)  - | 0.20  - | 0.69 (0.19-2.48)  - | 0.57 | 1.25 (0.51-3.08)  - | 0.62  - | 1.31 (0.57-3.03)  - | 0.51  - | 1.92 (0.80-4.59)  - | 0.14  - |
| Concurrent chemotherapy  Yes  No* | 0.07 (0.00-0.62)  - | **0.02**  - | 0.20 (0.02-1.80)  - | 0.15  - | 0.03 (0.00-0.37)  - | **0.006**  - | 0.11 (0.01- 0.87)  - | **0.04**  - | 0.03 (0.00-0.37)  - | **0.005^#^**  - |
| Median RT dose | 0.90 (0.83-0.97) | **0.009** | 0.98 (0.88-1.09) | 0.76 | 1.00 (0.95-1.04) | 0.90 | 0.96 (0.92-1.01) | 0.14 | 0.98 (0.94-1.02) | 0.27 |
| RT fractionation  Daily*  BID | -  1.87 (0.69-5.04) | -  0.22 | -  0.49 (0.17-1.39) | -  0.18 | -  0.93 (0.42-2.07) | -  0.87 | -  1.20 (0.57-2.54) | -  0.62 | -  1.00 (0.49-2.04) | -  0.99 |
| Tumor volume (cm^3^) | 1.00 (1.00-1.00) | 0.13 | 0.99 (0.98-1.00) | **0.007** | 1.00 (1.00-1.00) | 0.37 | 1.00 (1.00-1.00) | 0.10 | 1.00 (1.00-1.00) | 0.53 |
| Maximum 2D tumor diameter axial (cm) | 1.01 (1.00-1.02) | 0.44 | 1.10 (0.99-1.02) | 0.56 | 1.01 (0.99-1.03) | 0.14 | 1.01 (0.99-1.02) | 0.34 | 1.01 (0.99-1.02) | 0.48 |
| Maximum 2D tumor diameter coronal (cm) | 1.01 (1.00-1.02) | 0.14 | 1.00 (0.99-1.02) | 0.56 | 1.01 (1.00-1.02) | **0.03** | 1.01 (1.00-1.02) | **0.04** | 1.01  (0.99-1.02) | 0.03 |
| Maximum 2D tumor diameter sagittal (cm) | 1.01 (1.00-1.03) | 0.06 | 1.01 (1.00-1.03) | 0.19 | 1.01 (1.00-1.02) | 0.22 | 1.01 (1.00-1.02) | 0.14 | 1.00 (1.00-1.01) | 0.46 |
| Maximum 3D tumor diameter (cm) | 1.01 (1.00-1.02) | 0.16 | 1.01 (1.00-1.02) | 0.39 | 1.01 (1.00-1.02) | 0.12 | 1.01 (1.00-1.02) | 0.12 | 1.01 (1.00-1.01) | 0.29 |

*reference value; ^$^ Asian, not available and other

^#^Meets significance after adjustment for hypothesis testing using a Benjamini-Hochberg FDR of 0.1

**Supplemental Table 2. Multivariable Cox Analysis of predictors for Loco-regional recurrence (LRR), distant metastasis (DM), any progression and overall survival (OS) for patients with limited stage small cell lung cancer (LS-SCLC) treated with prophylactic cranial irradiation, (n=63)**

|  | **LRR** | | |
| --- | --- | --- | --- |
|  | **HR (95% CI)** | | **p-value** |
| Gender  Male | 0.37 (0.14-0.99) | | ***0.04^#^*** |
| N stage¥ | 0.01 (0.00-0.19) | | ***0.002^#^*** |
| Smoking£ | 8.27 (1.56-43.87) | | ***0.01^#^*** |
| Concurrent chemotherapy | 0.01 (0.00-0.06) | | ***<0.001^#^*** |
| Median RT dose | 0.84 (0.76-0.93) | | ***<0.001^#^*** |
|  |  | | |
|  | **In-field LRR** | | |
|  | **HR (95% CI)** | | **p-value** |
| Tumor volume (cm^3^) | 1.01 (1.00-1.02) | | ***0.04*** |
|  |  | | |
|  | **DM** | | |
|  | **HR (95% CI)** | **p-value** | |
| Gender  Male | 0.30 (0.12-0.72) | ***0.007^#^*** | |
| T stage* | 0.36 (0.14-0.96) | ***0.04^#^*** | |
| N stage¥ | 0.02 (0.01-0.32) | ***0.006^#^*** | |
| Performance status ≥ 1 | 3.42 (1.27-9.20) | ***0.01^#^*** | |
| Concurrent chemotherapy | 0.02 (0.01-0.27) | ***0.003^#^*** | |
| Induction chemotherapy | 0.43 (0.13-1.44) | 0.17 | |
| Maximum 2D tumor diameter coronal (cm) | 1.07 (1.00-1.03) | ***0.04^#^*** | |
|  | | | |
|  | **Any progression** | | |
|  | **HR (95% CI)** | | **p-value** |
| Gender  Male | 0.32 (0.15-0.66) | | ***0.002^#^*** |
| Age | 0.95 (0.90-1.01) | | 0.05 |
| Performance status ≥ 1 | 3.17 (1.36-7.41) | | ***0.008^#^*** |
| T stage* | 0.59 (0.26-1.34) | | 0.20 |
| N stage¥ | 0.06 (0.01-0.60) | | ***0.01^#^*** |
| Concurrent chemotherapy | 0.11 (0.01-1.08) | | 0.06 |
| Maximum 2D tumor diameter sagittal (cm) | 1.00 (0.99-1.01) | | 0.98 |
|  | | | |
|  | **OS** | | |
|  | **HR (95% CI)** | **p-value** | |
| Age | 0.96 (0.91-1.01) | 0.09 | |
| Gender  Male | 0.17 (0.08-0.36) | ***<0.001^#^*** | |
| T stage* | 0.28 (0.11-0.66) | ***0.004^#^*** | |
| N stage¥ | 0.02 (0.01-0.33) | ***0.004^#^*** | |
| Performance status ≥ 1 | 7.41 (2.81-19.50) | ***<0.001^#^*** | |
| RT fractionation | 0.43 (0.20-0.93) | ***0.03^#^*** | |
| Concurrent chemotherapy | 0.02 (0.01-0.27) | ***0.003^#^*** | |
| Maximum 2D tumor diameter coronal (cm) | 1.01 (0.99-1.02) | 0.20 | |

**^^^**Stage IA-IIIB as referent

*T stage 0-1 as referent

¥N stage 0-1 as referent

£Smoking: current + former as referent

^#^Meets significance after adjustment for hypothesis testing using a Benjamini-Hochberg FDR of 0.1

**Supplemental Table 3. Univariate Analysis of predictors for Loco-regional recurrence (LRR), distant metastasis (DM), any progression and overall survival (OS) for patients with limited stage small cell lung cancer (LS-SCLC) treated without prophylactic cranial irradiation, (n=42)**

| **Category** | **LRR** | | **In-field LRR** | | **DM** | | **Any progression** | | **OS** | |
| --- | --- | --- | --- | --- | --- | --- | --- | --- | --- | --- |
|  | **HR (95% CI)** | **p-value** | **HR (95% CI)** | **p-value** | **HR (95% CI)** | **p-value** | **HR (95% CI)** | **p-value** | **HR (95% CI)** | **p-value** |
| Gender  Female*  Male | -  2.25 (0.63-8.02) | -  0.21 | -  2.25 (0.26-18.82) | -  0.46 | -  0.96 (0.44-2.12) | -  0.92 | -  1.28 (0.59-2.76) | -  0.53 | -  0.63 (0.32-1.24) | -  0.18 |
| Age (continuous) | 0.99 (0.93-1.04) | 0.61 | 0.97 (0.89-1.06) | 0.51 | 0.96 (0.92-1.01) | 0.12 | 0.97 (0.93-1.02) | 0.22 | 0.98 (0.95-1.02) | 0.40 |
| Race  White*  African American  Other^$^ | -  1.39 (0.18-10.87)  0.50 (0.06-3.88) | -  0.75  0.51 | -  -  - | -  0.99  0.96 | -  1.21 (0.28-5.19)  0.50 (0.12-2.17) | -  0.80  0.36 | -  0.99 (0.23-4.22)  0.43 (0.10-1.82) | -  0.99  0.25 | -  1.16 (0.27-4.89)  0.64 (0.19-2.11) | -  0.84  0.46 |
| Performance score  0*  ≥1 | -  1.77 (0.40-7.89) | -  0.46 | -  0.52 (0.10-2.75) | -  0.44 | -  2.38 (0.69-8.15) | -  0.17 | -  2.72 (0.81-9.18) | -  0.11 | -  2.26 (0.79-6.46) | -  0.13 |
| Smoking history  Current*  Former  Never | -  0.36 (0.12-1.06)  - | -  0.06  - | -  1.14 (0.21-5.94)  - | -  0.88  - | -  0.72 (0.33-1.56)  - | -  0.41  - | 0.67 (0.32-1.40)  - | -  0.28  - | -  0.85 (0.43-1.65)  - | -  0.63  - |
| Pack-years | 1.01 (0.99-1.02) | 0.36 | 1.00 (0.99-1.02) | 0.12 | 1.01 (1.00-1.02) | 0.79 | 1.01 (0.99-1.01) | 0.81 | 1.01 (1.00-1.01) | 0.91 |
| T stage  T0-1*  T2-4 | -  0.76 (0.27-2.11) | -  0.59 | -  0.29 (0.06-1.53) | -  0.15 | -  0.85 (0.38-1.86) | -  0.68 | -  0.78 (0.37-1.63) | -  0.51 | -  0.96 (0.48-1.88) | -  0.91 |
| N stage  N0-1*  N2-3 | -  2.25 (0.28-17.67) | -  0.44 | -  - | -  - | -  1.46 (0.34-6.22) | -  0.61 | -  1.78 (0.42-7.55) | -  0.43 | -  1.63 (0.49-5.45) | -  0.43 |
| Overall stage  IIIB*  IA-IIIA  IIIC | -  -  4.30  (1.20-15.82) | -  -  **0.03** | -  -  - | -  ^-^  - | -  1.23 (0.26-5.71)  1.37 (0.59-3.17) | -  0.79  0.46 | -  1.07 (0.23-4.89)  1.57 (0.71-3.49) | -  0.93  0.25 | -  0.99 (0.22-4.51)  1.58 (0.75-3.31) | -  1.00  0.22 |
| Induction chemotherapy  Yes  No* | 0.91 (0.20-4.07)  - | 0.90  - | 0.58 (0.07-5.04)  - | 0.63 | 0.99 (0.29-3.28)  - | 0.98  - | 1.09 (0.33-3.62)  - | 0.89  - | 0.87 (0.33-2.25)  - | 0.77  - |
| Concurrent chemotherapy  Yes  No* | 0.63 (0.19-1.98)  - | 0.43  - | 1.55 (0.18-13.31)  - | 0.69  - | 0.83 (0.33-2.08)  - | 0.70  - | 0.61 (0.27- 1.38)  - | 0.24  - | 0.58 (0.28-1.22)  - | 0.15  - |
| Median RT dose | 1.01 (0.95-1.07) | 0.70 | 1.00 (0.89-1.10) | 0.93 | 1.04 (1.00-1.09) | 0.13 | 1.04 (0.99-1.08) | 0.13 | 1.01 (0.99-1.05) | 0.55 |
| RT fractionation  Daily*  BID | -  0.72 (0.20-2.58) | -  0.61 | -  1.29 (0.25-6.80) | -  0.76 | -  1.10 (0.44-2.76) | -  0.83 | -  0.85 (0.35-2.10) | -  0.73 | -  0.89 (0.40-1.99) | -  0.79 |
| Tumor volume (cm^3^) | 1.00 (1.00-1.00) | 0.74 | 1.00 (1.00-1.00) | 0.83 | 1.00 (1.00-1.00) | 0.74 | 1.00 (1.00-1.00) | 0.61 | 1.00 (1.00-1.00) | 0.72 |
| Maximum 2D tumor diameter axial (cm) | 1.01 (1.00-1.02) | **0.04** | 1.00 (0.90-1.03) | 0.65 | 1.00 (0.99-1.01) | 0.51 | 1.01 (0.99-1.01) | 0.22 | 1.01 (0.99-1.01) | 0.28 |
| Maximum 2D tumor diameter coronal (cm) | 1.01 (1.00-1.01) | 0.06 | 1.01 (1.00-1.03) | 0.19 | 1.00 (1.00-1.01) | 0.50 | 1.00 (1.00-1.01) | 0.35 | 1.00  (1.00-1.01) | 0.35 |
| Maximum 2D tumor diameter sagittal (cm) | 1.00 (0.99-1.02) | 0.13 | 1.00 (0.99-1.02) | 0.65 | 1.00 (0.99-1.01) | 0.47 | 1.00 (0.99-1.01) | 0.43 | 1.00 (1.00-1.01) | 0.53 |
| Maximum 3D tumor diameter (cm) | 1.02 (1.00-1.03) | **0.02** | 1.02 (0.99-1.05) | 0.17 | 1.01 (1.00-1.01) | 0.15 | 1.01 (0.99-1.02) | 0.07 | 1.01 (1.00-1.02) | 0.10 |

*reference value; ^$^ Asian, not available and other

^#^Meets significance after adjustment for hypothesis testing using a Benjamini-Hochberg FDR of 0.1

**Supplemental Table 4. Univariate Analysis of predictors for Loco-regional recurrence (LRR), distant metastasis (DM), any progression and overall survival (OS) for patients with limited stage small cell lung cancer (LS-SCLC) treated with chemoradiation, (n=105)**

| **Category** | **LRR** | | **In-field LRR** | | **DM** | | **Any progression** | | **OS** | |
| --- | --- | --- | --- | --- | --- | --- | --- | --- | --- | --- |
|  | **HR (95% CI)** | **p-value** | **HR (95% CI)** | **p-value** | **HR (95% CI)** | **p-value** | **HR (95% CI)** | **p-value** | **HR (95% CI)** | **p-value** |
| Gender  Female*  Male | -  0.75 (0.39-1.44) | -  0.39 | -  0.80 (0.38-1.71) | -  0.57 | -  0.70 (0.41-1.21) | -  0.20 | -  0.70 (0.43-1.16) | -  0.17 | -  0.50 (0.32-0.80) | -  **0.003^#^** |
| Age (continuous) | 0.97 (0.94-1.01) | 0.20 | 0.95 (091-0.99) | **0.04** | 1.00 (0.97-1.03) | 0.96 | 0.99 (0.96-1.02) | 0.56 | 1.01 (0.98-1.04) | 0.46 |
| Race  White*  African American  Other^$^ | -  0.65 (0.09-4.76)  0.37 (0.05-2.67) | -  0.67  0.32 | -  -  0.47 (0.06-3.74) | -  0.99  0.46 | -  1.44 (0.45-4.64)  0.89 (0.28-2.86) | -  0.54  0.84 | -  1.22 (0.38-3.89)  0.71 (0.22-2.28) | -  0.74  0.60 | -  1.31 (0.47-3.66)  1.00 (0.36-2.76) | -  0.61  0.99 |
| Performance score  0*  ≥1 | -  2.00 (0.94-4.25) | -  0.07 | -  1.61 (0.71-3.66) | -  0.25 | -  2.92 (1.46-5.84) | -  **0.002^#^** | -  2.90 (1.53-5.48) | -  **0.001^#^** | -  3.05 (1.70-5.45) | -  **<0.001^#^** |
| Smoking history  Current*  Former  Never | -  0.71 (0.36-1.39)  1.92 (0.45-8.19) | -  0.32  0.38 | -  -  - | -  -  - | -  1.05 (0.61-1.79)  0.52 (0.07-3.83) | -  0.87  0.52 | 0.99 (0.60-1.65)  1.16 (0.28-4.81) | -  0.97  0.84 | -  1.15 (0.72-1.82)  0.99 (0.24-4.1) | -  0.56  0.99 |
| Pack-years | 1.01 (0.99-1.02) | 0.21 | 1.00 (0.99-1.02) | 0.96 | 1.01 (1.00-1.02) | 0.11 | 1.01 (0.99-1.01) | 0.20 | 1.01 (1.00-1.02) | 0.12 |
| T stage  T0-1*  T2-4 | -  0.66 (0.34-1.28) | -  0.22 | -  0.93 (0.44-1.99) | -  0.85 | -  0.64 (0.37-1.12) | -  0.12 | -  0.64 (0.38-1.08) | -  0.09 | -  0.67 (0.41-1.10) | -  0.11 |
| N stage  N0-1*  N2-3 | -  5.47 (0.75-39.93) | -  0.09 | -  - | -  - | -  1.64 (0.59-4.56) | -  0.34 | -  2.06 (0.75-5.67) | -  0.16 | -  1.35 (0.62-2.95) | -  0.45 |
| Overall stage  IIIB*  IA-IIIA  IIIC | -  0.59 (0.28-1.25)  1.34  (0.61-2.95) | -  0.17  0.47 | -  0.32 (0.13-0.78)  0.33 (0.12-0.94) | -  **0.01^#^**  **0.04** | -  0.68 (0.36-1.28)  1.45 (0.75-2.78) | -  0.23  0.26 | -  0.69 (0.39-1.22)  1.37 (0.73-2.55) | -  0.20  0.33 | -  0.73 (0.44-1.22)  0.97 (0.54-1.79) | -  0.23  0.95 |
| Induction chemotherapy  Yes  No* | 1.77 (0.69-4.53)  - | 0.24  - | 1.67 (0.58-4.80)  - | 0.34 | 1.33 (0.65-2.73)  - | 0.43  - | 1.43 (0.73-2.82)  - | 0.30  - | 1.65 (0.87-3.14)  - | 0.12  - |
| Concurrent chemotherapy  Yes  No* | 0.43 (0.17-1.11)  - | 0.08  - | 0.89 (0.21-3.77)  - | 0.88  - | 0.40 (0.18-0.90)  - | **0.03**  - | 0.33 (0.16- 0.68)  - | **0.003^#^**  - | 0.29 (0.15-0.54)  - | **<0.001^#^**  - |
| PCI  Yes  No* | 0.71 (0.37-1.36)  - | 0.30  - | 1.21 (0.53-2.74)  - | 0.65  - | 0.40 (0.23-0.68)  - | **<0.001^#^**  - | 0.44 (0.27-0.73)  - | **0.001^#^**  - | 0.37 (0.23-0.59)  - | **<0.001^#^**  - |
| Median RT dose | 0.97 (0.93-1.01) | 0.15 | 0.94 (0.90-0.99) | **0.02^#^** | 1.03 (1.00-1.06) | **0.04** | 1.01 (0.99-1.04) | 0.34 | 1.01 (0.99-1.04) | 0.34 |
| RT fractionation  Daily*  BID | -  1.13 (0.59-2.13) | -  0.72 | -  1.49 (0.70-3.16) | -  0.30 | -  0.68 (0.40-1.17) | -  0.16 | -  0.76 (0.46-1.26) | -  0.29 | -  0.64 (0.40-1.02) | -  0.06 |
| Tumor volume (cm^3^)  0.2-48.5*  48.6-428 | -  1.28 (0.67-2.43) | -  0.44 | -  1.30 (0.62-2.75) | -  0.49 | -  1.01 (0.59-1.71) | -  0.97 | -  1.15 (0.70-1.89) | -  0.58 | -  0.96 (0.61-1.51) | -  0.86 |
| Maximum 2D tumor diameter axial (cm)  1-7.4*  7.5-15.1 | -  1.24 (0.65-2.33) | -  0.51 | -  0.61 (0.27-1.36) | -  0.23 | -  1.34 (0.79-2.28) | -  0.28 | -  1.30 (0.79-2.13) | -  0.30 | -  1.23 (0.78-1.94) | -  0.37 |
| Maximum 2D tumor diameter coronal (cm)  0.6-8.6*  8.7-21.6 | -  1.55 (0.82-2.93) | -  0.18 | -  1.47 (0.69-3.12) | -  0.32 | -  1.58 (0.92-2.69) | -  0.09 | -  1.58 (0.96-2.6) | -  0.07 | -  1.51 (0.96-2.38) | -  0.07 |
| Maximum 2D tumor diameter sagittal (cm)  0.9-8.3*  58.4-20.7 | -  1.62 (0.84-3.10) | -  0.15 | -  0.85 (0.39-1.83) | -  0.67 | -  1.03 (0.60-1.74) | -  0.92 | -  1.03 (0.63-1.69) | -  0.91 | -  0.93 (0.59-1.48) | -  0.77 |
| Maximum 3D tumor diameter (cm)  1-10.8*  10.9-22.2 | -  2.19 (1.13-4.23) | -  **0.02** | -  0.96 (0.44-2.07) | -  0.91 | -  1.64 (0.96-2.79) | -  0.07 | -  1.61 (0.97-2.64) | -  0.06 | -  1.29 (0.82-2.04) | -  0.26 |

*reference value; ^$^ Asian, not available and other

^#^Meets significance after adjustment for hypothesis testing using a Benjamini-Hochberg FDR of 0.1
